# Stimulation of Synthesis and Lysis of Extracellular Matrix Proteins in Fibrosis Associated with Lymphedema

**DOI:** 10.3390/dermatopathology9010001

**Published:** 2021-12-28

**Authors:** Jose Maria Pereira de Godoy, Maria de Fatima Guerreiro Godoy, Henrique Jose Pereira de Godoy, Dalisio De Santi Neto

**Affiliations:** 1 Department of Cardiology and Cardiovascular Surgery, São José do Rio Preto School of Medicine (FAMERP), CNPq (National Council for Research and Development), Floriano Peixoto, Sao Jose do Rio Preto 15020-010, Brazil; 2 Postgraduate Program Stricto Sensu, São José do Rio Preto School of Medicine (FAMERP), Sao Jose do Rio Preto 15020-010, Brazil; mfggodoy@gmail.com; 3 Research Group at Godoy Clinic, Sao Jose do Rio Preto 15020-010, Brazil; henriquegodoy95@gmail.com; 4 Department of General Surgery, São José do Rio Preto School of Medicine (FAMERP), Sao Jose do Rio Preto 15020-010, Brazil; 5 Hospital Affiliated with São José do Rio Preto School of Medicine (FAMERP), Sao Jose do Rio Preto 15020-010, Brazil; dalisio@gmail.com

**Keywords:** extracellular matrix, histological reversal, lysis, physiological stimulation, proteins, synthesis

## Abstract

*Background*: Fibrotic diseases pose a problem for overall health due to their chronic, progressive nature; the lack of a cure; and the fact that such conditions are largely refractory to current medical and surgical treatment practices. *Objective*: The aim of the present study was to report the physiological stimulation of synthesis and lysis of extracellular matrix proteins during the treatment of primary lymphedema. *Material and Methods*: A clinical trial was conducted involving the analysis of changes in type I and III collagen fibers and elastic fibers as well as the thickness of the epidermis and dermis in 10 histological fields. Samples were taken from the skin before and after intensive treatment using the Godoy Method^®^ and adapted to the treatment of fibrosis in a patient with a clinical diagnosis of lower limb lymphedema. Slides were stained with orcein, hematoxylin and eosin, picrosirius red, and Gomori’s reticulin stains. Weibel’s multipoint method was used for the morphometric evaluation. The data were compared using the *t*-test with a 95% confidence interval. *Results*: Significant changes were detected in all aspects of interest (thickness of the epidermis and dermis, type I and III collagen fibers, and elastic fibers). *Conclusion*: The present findings demonstrate the physiological stimulation of synthesis and lysis of the main components of an extracellular matrix, such as type I and III collagen fibers and elastic fibers, as well as a reduction in the thickness of the epidermis and dermis in cases of fibrosis through adequate stimulation of the lymphatic system.

## 1. Introduction

Fibrotic diseases pose a problem for overall health due to the lack of a cure and the chronic, progressive nature, affecting organs of the body in an isolated manner or systemically. Such conditions are largely refractory to current medical and surgical treatment practices [1]. Fibrosis is believed to contribute to about 45% of deaths in the industrialized world [2].

Lymphedema is a condition involving the accumulation of macromolecules in the interstitial space in skin and subcutaneous tissue, causing important changes to the components of an extracellular matrix (ECM), especially collagen fibers, elastin, fibroblast activation, and keratocytes [3,4]. During the matrix formation phase, there is a disproportional accumulation of collagen and other ECM proteins, which impedes the restoration of the architecture and normal functioning of the tissue. The fibrotic repair process is extraordinarily complex and involves a broad spectrum of cells, signaling pathways, and regulatory systems, some of which may be abruptly interrupted, contributing to fibrotic lesions [5].

Histopathological and immunohistochemical analyses of nodules in patients with lymphedema reveal an increase in type I and III collagen in comparison with normal skin. Gene transcript analysis reveals a significant positive regulation of type III collagen versus type I collagen [6]. Regardless of the initial events, the activation of ECM-producing cells is a common characteristic of all fibrotic conditions.

In recent years, novel concepts have emerged in the treatment of lymphedema, with the proposal of the normalization or near normalization of the affected limb in all clinical stages, including elephantiasis, and this treatment modality has been adapted to the clinical reversal of fibrosis [7,8,9,10,11,12,13,14,15]. Intensive treatment eight hours per day for one week enables the reduction to the volume of the lymphedema by around 50% in five days. This treatment consists of a combination of therapies [8,9,10,11] performed intensively or adapted to each patient [12,13,14,15]. Normalization is achieved when the volume of the affected limb resembles that of the unaffected contralateral limb (evaluated by volumetry) and the elasticity of the skin returns (evaluated by the manual pinching of the skin).

The aim of the present study was to report on physiological stimulation of the synthesis and lysis of ECM proteins during the clinical treatment of primary lymphedema adapted to the reversal of fibrosis.

## 2. Material and Methods

### 2.1. Setting

A patient with a clinical diagnosis of lower limb lymphedema visited the vascular surgery outpatient clinic of the University Hospital affiliated with the Faculdade de Medicina de Sao Jose do Rio Preto (FAMERP (Sao Jose do Rio Preto School of Medicine), Brazil; the Godoy Clinic in the city of Sao Jose do Rio Preto, Brazil; and the Microscopy and the Microanalysis Multiuse Center of the Biology Department of the Institute of Biosciences, Letters, and Exact Sciences of Universidade Estadual Paulista (UNESP, São Jose do Rio Preto Campus), in 2020.

### 2.2. Design

A clinical trial was conducted involving the analysis of changes in type I and III collagen fibers and elastic fibers as well as changes in the thickness of the epidermis and dermis in 10 histological fields. Samples were taken from the skin before and after intensive treatment with the Godoy method, adapted to the treatment of fibrosis. The selection of samples was based on random blade material. Weibel’s multipoint method was used for the morphometric evaluation. The data were compared using the paired *t*-test with a 95% confidence interval.

### 2.3. Inclusion Criterion

The inclusion criterion was stage II lower limb lymphedema with intense fibrosis and the absence of Godet’s sign.

### 2.4. Exclusion Criteria

The exclusion criteria included active infection or edema due to other causes, diagnosed clinically.

### 2.5. Statistical Analysis

The data were submitted to a normality test and expressed as mean and standard deviation values. Comparisons were performed using the *t*-test with the level of significance set at 95%.

### 2.6. Presentation

**Figure 1:**
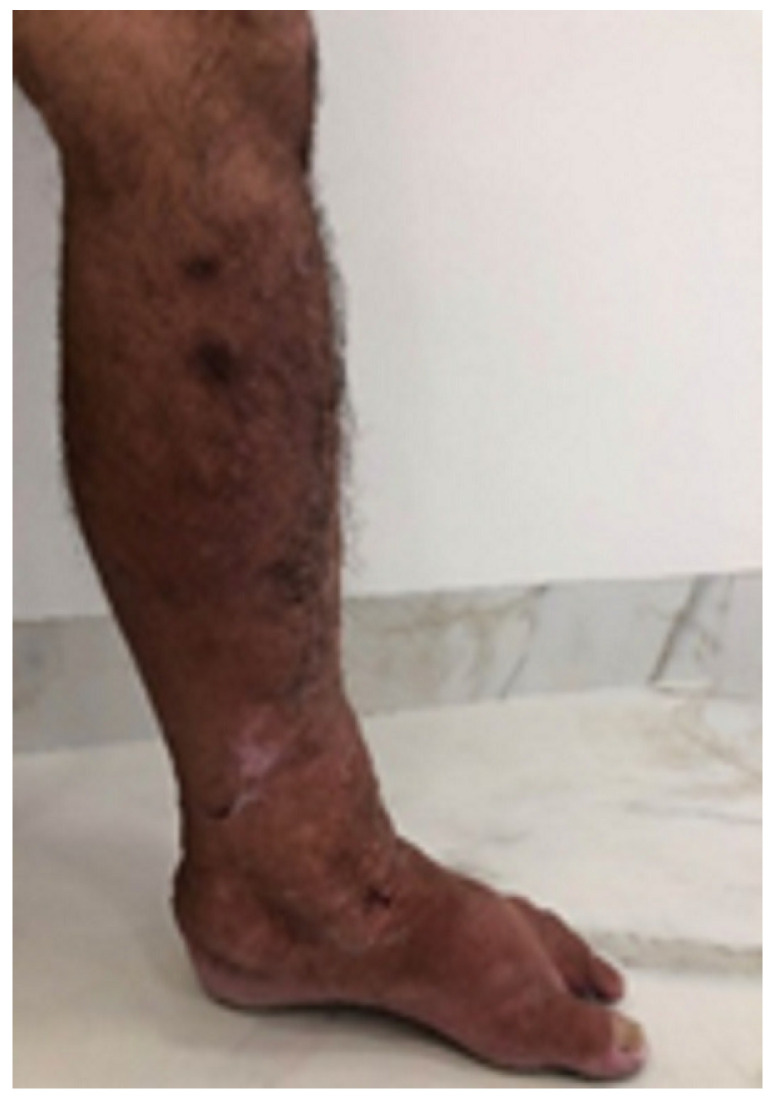
Limb with stage II lymphedema and biopsy site.

**Figure 2:**
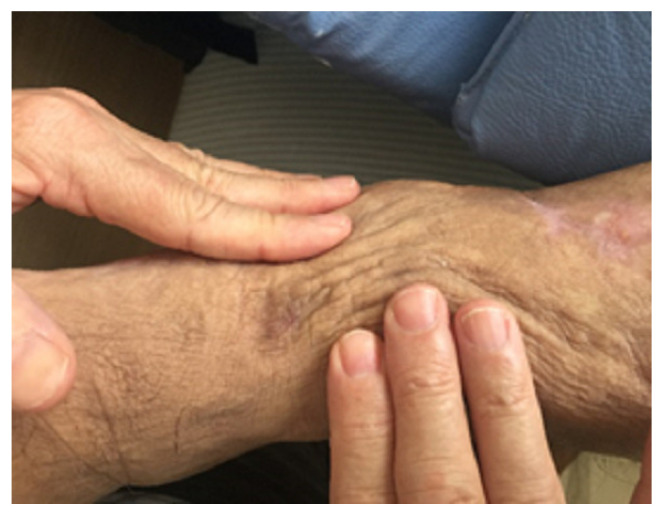
Limb after treatment, with clinical reversal of fibrosis and improvement in elasticity of the skin.

A 67-year-old male patient with late-onset primary lymphedema diagnosed at 55 years of age and one episode of erysipelas in this 12-year period agreed to participate in the study and signed a statement of informed consent. Other causes of clinical edema, such as kidney disease, heart disease, hypoproteinemia, and chronic venous disease, were discarded. The patient underwent a physical examination, which revealed intense fibrosis of the dermis characterized by the absence of Godet’s sign. The volumetric analysis involved the water displacement method, which revealed that the affected limb weighed 1200 g more than the contralateral limb. Photographs and video images were taken before and after treatment. A biopsy was performed in the inframalleolar region of the left leg (Figure 1). After clinical normalization of the fibrosis and the return of elasticity, a second biopsy was performed 0.5 cm parallel and lateral to the first incision (Figure 2). For the biopsies, basic surgical care was taken, such as asepsis and antisepsis, local anesthesia using 2 mL of xylocaine 2%, followed by a longitudinal wedge-shaped incision approximately 1 cm in length and 0.5 cm in width. The biopsied material was maintained in 10% formol and embedded in paraffin. The slides were stained with orcein, hematoxylin and eosin, picrosirius red, and Gomori’s reticulin, and evaluated under an optical microscope. Ten randomly selected histological fields were evaluated using the multipoint morphometric method proposed by Weibel (1964).

### 2.7. Treatment

The Godoy intensive treatment method [7] was performed, which consists of cervical lymphatic therapy using the Godoy method (approximately 30 gentle movements on the skin in the supraclavicular region 15 to 20 min per day) [8], combined with eight hours of mechanical lymphatic therapy involving an electromechanical device that performs approximately 25 passive plantar flexion and extension movements per minute [9], two hours per day of manual lymphatic therapy involving linear movements toward the corresponding lymph nodes [10], and a compression mechanism (hand-crafted stocking made with grosgrain fabric [11] alternated with medium-stretch elastic bandages maintained throughout the entire treatment). The duration of treatment was two months, when the clinical reversal of fibrosis was achieved and the elasticity of the skin improved. At this point, the post-intervention biopsy was performed.

## 3. Results

**Figure 3:**
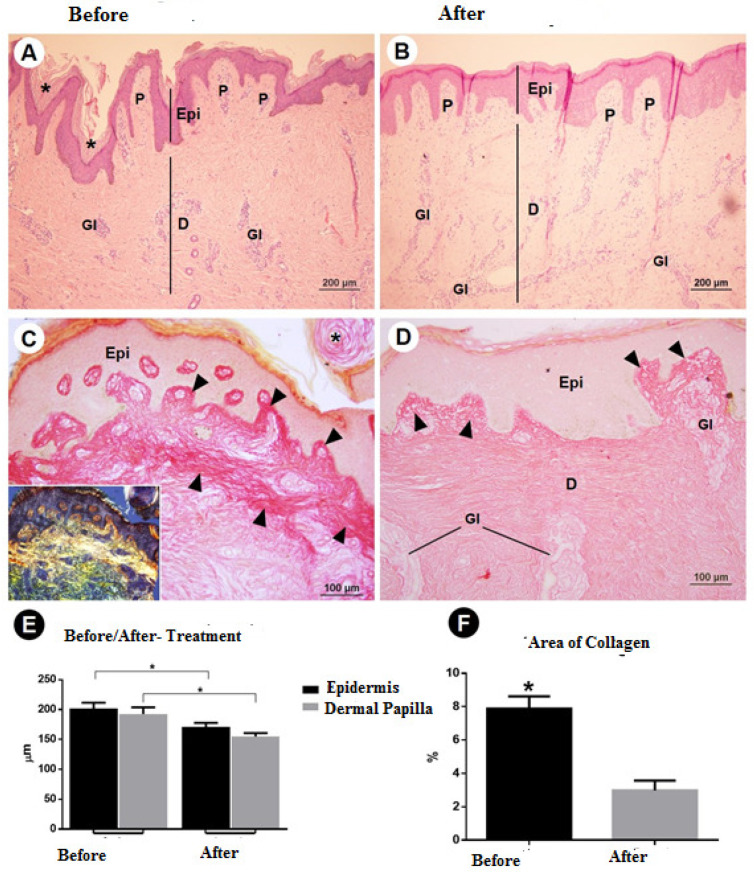
Morphometry of the skin and type I collagen fibers. (**A**,**B**) Epidermis (Epi) and dermis (D) before and after treatment. Skin with irregular surface, increased thickness of epidermis, irregular dermal papillae, and increase in papilla height before treatment. (**C**,**D**) Type I collagen was denser in the dermis before treatment (inset), but was reduced in the collagen area and restricted to region of papillae after treatment. (**E**) Morphometry of epidermis and dermal papillae. Note the significant difference between pre-treatment and post-treatment. (**F**) Percentage of area with type I collagen in the dermis. Note the reduction, with a significantly greater area prior to treatment (asterisk*). Abbreviations and symbols: Asterisks (*), regions of hyperkeratosis; Epi, epidermis; D, dermis; Gl, dermal glands; P, dermal papilla. Stains: A, B: HE; C, D: picrosirius red; inset: picrosirius under polarized light.

**Figure 4:**
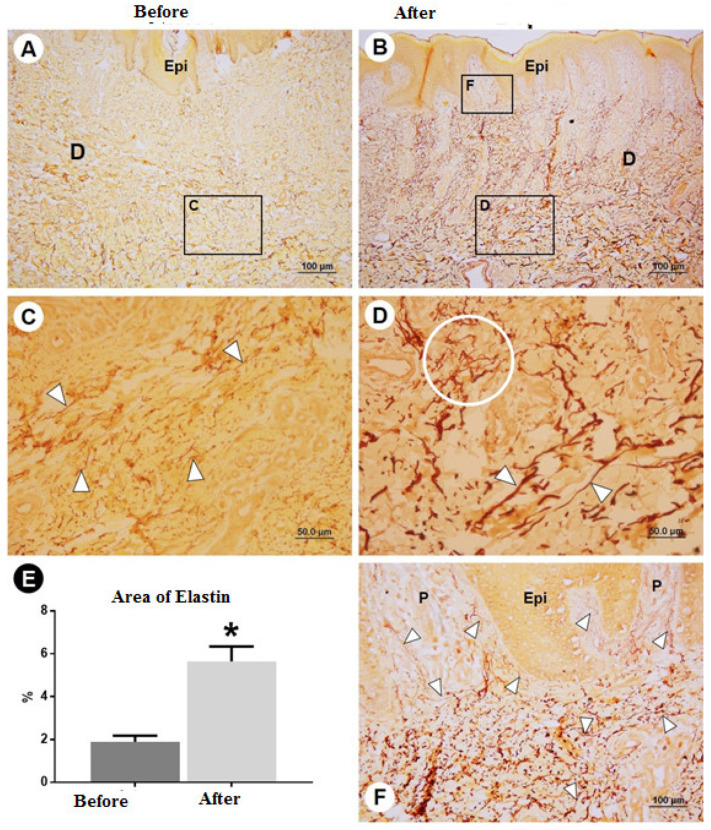
Elastic fibers. (**A**,**B**) Elastin in papillary and reticular strata of the dermis, with greater density after treatment. (**C**,**D**) Elastic fibers are thin and in small quantity before treatment, fibers in the deep dermis thickened and increased in quantity after treatment. (**E**) Percentage of elastin in dermis before and after treatment. Note the significant increase in fibers after treatment (asterisk *) attributable to the synthesis of new elastic fibers, as shown in (**F**). Abbreviations and symbols: asterisks (*), fibers after treatment; Epi, epidermis; D, dermis; P, dermal papilla; arrowheads (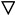) elastic fibers. Stain: orcein.

**Figure 5:**
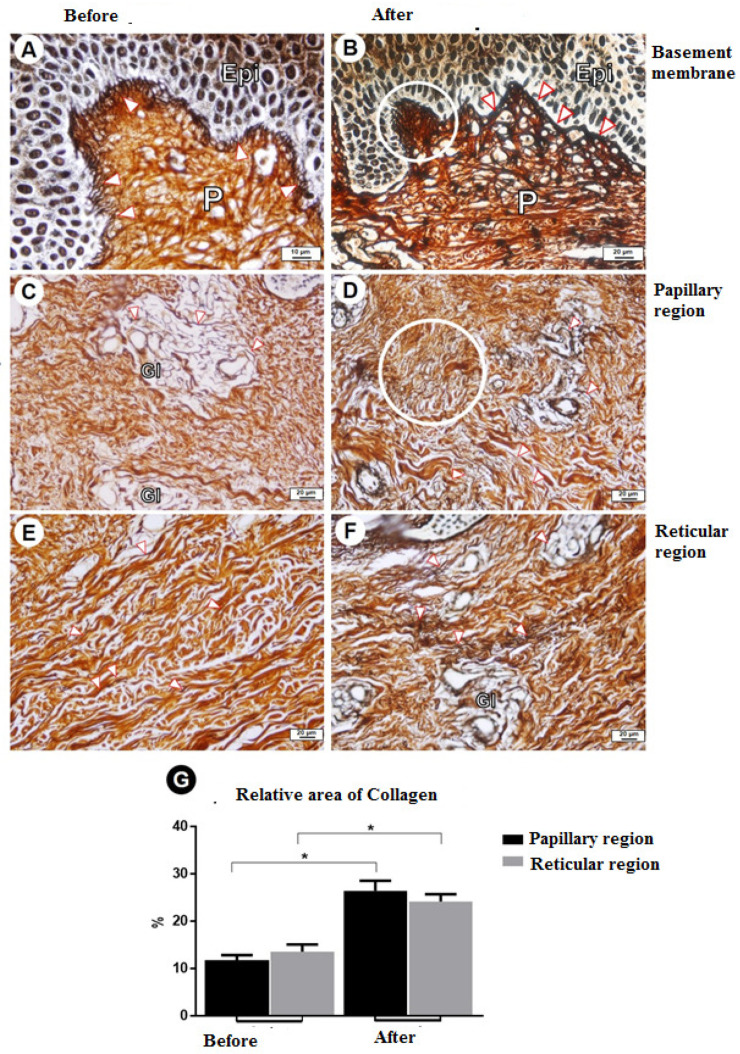
Reticular fibers (collagen III). (**A**,**B**) Region of basement membrane (epidermis–papillary dermis interface) with ruptured reticular fibers of low density before treatment; thickening of fibers; continuity (non-rupture) of fibers in basement membrane after treatment; and increase in these fibers in dermal papillae, forming a collagen III network. (**C**,**D**) Deposition of collagen III in the papillary region. Note the fibers restricted to glandular and peri-glandular regions before treatment and found in the entire compartment after treatment. (**E**,**F**) Reticular region of dermis. Note the collagen III fibers before treatment and relative increase in and thickening of fibers after treatment. (**G**) Bar chart shows a significant increase in the relative area of collagen III in different strata of the dermis after treatment. *p* value < 0.05. Abbreviations and symbols: Epi, epidermis; P, dermal papilla; Gl, dermal glands: red arrowheads (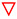), reticular fibers. Stain: Gomori’s reticulin. Asterisks *, regions of hyperkeratosis.

The clinical evaluation revealed a clear improvement in the elasticity of the skin after treatment, as shown in Figure 1 and Figure 2. The morphometric analysis revealed a significant change in all aspects of interest, with a reduction in the thickness of the epidermis and dermis, an increase in elastin, a reduction in type I collagen, and an increase in type III collagen (Table 1). Figure 3 shows the morphometric findings of the skin and type I collagen fibers before and after treatment. The slides were stained with hematoxylin and eosin and with picrosirius red. Prior to treatment, the skin had an irregular surface, with increased thickness of the epidermis, irregular dermal papillae, increased papilla height, and denser type I collagen in the dermis. After treatment, a reduction was found in the area of collagen, which was restricted to the region of the papillae, and significant reductions were found in the epidermis and dermal papillae. Figure 4 shows the changes in elastic fibers, evidenced by orcein staining. Prior to treatment, the elastic fibers were thin and in smaller quantities in the dermis. After treatment, the fibers were thicker in the deep dermis and in greater quantities. A greater density of elastin was found in the strata of the dermis (papillary and reticular regions) after treatment, and a substantial increase in these fibers was found, which could be attributed to the synthesis of new elastic fibers. Figure 5 shows the reticular fibers (collagen III) before and after treatment, stained with Gomori’s reticulin. The region of the basement membrane (epidermis–papillary dermis interface) exhibited ruptured reticular fibers with low density before treatment, with the thickening and continuity (absence of rupture) of these fibers in the basement membrane after treatment. An increase in these fibers was found in the dermal papillae, forming a collagen III network. Prior to treatment, these fibers were restricted to the glandular and pre-glandular regions, whereas the fibers were found throughout the entire compartment after treatment. In the reticular region of the dermis, collagen III fibers were found prior to treatment, whereas a relative increase and thickening of these fibers were found after treatment. The bar chart in the Figure 5 shows the significant increase in the relative area of collagen III in the different regions of the dermis after treatment.

## 4. Discussion

The present study demonstrated the clinical reversal of fibrosis through specific physiological stimulation of the lymphatic system, resulting in the synthesis and lysis of the main proteins of an extracellular matrix in the skin of a patient with primary lymphedema. The gold standard method of evaluation (histopathology) was employed before and after treatment, and quantification was performed using morphometry. No previous studies in the literature have presented similar findings. Therefore, the present investigation offers an important discovery on how to stimulate the synthesis and lysis of ECM proteins.

The aspects of interest in this study were type I and III collagen fibers, elastic fibers, and the thicknesses of the dermis and epidermis. A significant reduction in collagen I fibers by more than 250% was found, with remodeling and redistribution of these fibers in the dermis. An increase in collagen III fibers of around 100% was found in both the papillary and reticular regions. Moreover, an increase in elastic fibers of around 250% was found, with remodeling and redistribution of these fibers in the dermis. Significant reductions also occurred in the thickness of the epidermis and dermis. These changes suggest that the method employed may affect other collagen fibers, paving the way for an important line of research in the treatment of diseases with inflammatory and fibrotic characteristics.

A study involving the histological analysis of biopsies of nodules from the limbs of patients with stage III lymphedema detected increases in type I and III collagen in the semiquantitative analysis (immunohistochemical analysis with hematoxylin-DAB staining) compared with normal skin, and the gene transcript analysis revealed a significant up-regulation of type III collagen vs. type I collagen [6]. The treatment performed in the present investigation led to an important reduction in type I collagen of around 250% and a 100% increase in type III collagen. Another study by the authors comparing the normal limb before and after treatment is in the analysis phase.

A study analyzing skin biopsies at different stages of lymphedema due to filaria detected an increase in perivascular fibrosis in stages 2 and 3, which was more prevalent in the deep dermis than the superficial dermis. An increase was found in mononuclear cells from 50% in stage 1 to 90% in stage 2. Participation in a lymphedema management program for one year was associated with reductions in cellular infiltrate and fibrosis, suggesting that basic care can reduce inflammatory processes in patients with lymphedema caused by filaria [12].

The literature offers few data on the histological evaluation of lymphedema but reports important inflammatory processes in filariasis, which should be evaluated better in cases of primary lymphedema. Different physiopathological processes may be involved in the development of lymphedema. However, stimulation of the lymphatic system seems to be the most common mode of treatment and reversal in such cases. One study showed that lymphovenous surgery achieved important clinical improvements in a patient with chronic venous ulcer, signs of lymphatic impairment, and lymphorrhagia [16]. Another study showed that the lymphatic capillary network is destroyed in more severe stages of chronic venous insufficiency and that the remaining capillaries have increased permeability, suggesting an additional lymphatic component in the formation of edema [17].

The present study was conducted to confirm clinical findings in the treatment of lymphedema, with the achievement of normality or near normality in all clinical stages, including clinical stage III (elephantiasis) with the Godoy treatment method. In cases of stage II lymphedema, a deficiency in the formation or drainage of lymph nodes is seen. The physiopathological mechanism involved is the accumulation of macromolecules and fluid in the interstitial space that could progress to a condition of intense fibrosis, denoting initial changes in the fundamental substance with the progression to changes in ECM proteins. The treatment employed in the present study interferes in the physiopathology of such cases, mobilizing macromolecules through the lymphatic system. Thus, the therapeutic strategy for a reversal of various causes of fibrotic processes involves interference in the physiopathology of each disease.

Homeostasis of the ECM involves a complex mechanism of the synthesis and lysis of its components, in which diverse chemical and mechanical stimuli, such as cytokines, growth factors, proteases, lipid mediators, and mechanical forces, play important roles [4]. The literature reports several mechanisms involved in this process, and the identification of each mechanism can lead to therapeutic advances with regard to different diseases.

The treatment employed in this study was the Godoy and Godoy method for the reversal of lymphedema in all clinical stages, including elephantiasis, to standards of normality or near normality, which was adapted to the reversal of fibrosis, confirming the importance of mobilization methods. The main mobilization pathway for macromolecules is the lymphatic system, but physiological lysis of the proteins is necessary. In the evolution of this process, other mechanisms involved in the production of ECM and the interference in gene expression, such as inflammatory processes, may occur.

The dynamics of the mobilization of macromolecules from the ECM to blood circulation almost mandatorily passes through the lymphatic system. This system is the functional reserve of the venous system, and edema occurs when this reserve is surpassed. Thus, this problem involves microcirculation, and both arterial and venous circulation can interfere in these dynamics.

Treating and curing fibrosis is difficult when the lymphatic system fails. To date, no cure exists, but improvements can be achieved, as we have demonstrated in our past publications [7,12,13,14,15]. We are able to bring the affected limb to within or close to normality and without fibrosis, but the condition is chronic and requires maintainenance of treatment. This is true for fibrosis caused by lymphedema. However, with other causes, such as inflammatory processes, fibrosis can be cured when the causal agent is eliminated. Therefore, this is the main treatment route to be encouraged.

The present investigation broadens the horizon of guiding the reversal of fibrosis. Therefore, we believe that one of the main lines of action is stimulation of the lymphatic system, which is responsible for the mobilization of nearly all macromolecules through interstitial and vascular (arterial, venous, and lymphatic) circulation. Thus, we stress the importance of the lymphatic system in the physiopathology of lymphedema.

This study provides the first evaluations that we performed, and is of fundamental importance in gaining a better understanding of the proteolytic process, the mechanisms involved, the changes in EMC-producing cells, and genetic interference in this important dynamic of the extracellular matrix. The use of scanning electronic microscopy and novel markers is currently being studied, and novel results will soon be available. Gaining a better understanding of the different stages and progression of fibrosis is also important.

## 5. Conclusions

The present findings demonstrate physiological stimulation of the synthesis and lysis of the main components of an extracellular matrix, such as type I and III collagen fibers and elastin, as well as a reduction in the thickness of the epidermis and dermis through adequate stimulation of the lymphatic system using a specific drainage method, resulting in the reversal of fibrosis.

## Figures and Tables

**Table 1:** Morphometric findings in epidermis and dermal papillae, and area of connective tissue fibers before and after treatment (SD and SEM).

		Before	After	
Morphometry	Epidermis (µm)	202 ± 9.52 a	171.2 ± 6.54 b	*p* < 0.05
	Dermal papilla (µm)	192.6 ± 11.23 a	155.1 ± 5.58 b	*p* < 0.05
Area of Collagen (%)		1.90 ± 0.19 b	5.652 ± 0.32 a	*p*< 0.001
Area of Elastic Fibers (%)		2.98 ± 0.68 b	7.88 ± 0.28 a	*p* < 0.001
Area of Collagen III (%)	Papillary region	11.77 ± 1.12 b	26.46 ± 2.11 a	*p* < 0.05
	Reticular region	13.61 ± 1.51 b	24.23 ± 1.50 a	*p* < 0.05

a, b different letters denote significant difference between groups (*p* < 0.05). SD: standard deviation; SEM ±: standard error of mean.

## Data Availability

The data used to support the findings of this study are included within the article.
